# Preparation of Mesoporous and/or Macroporous SnO_2_-Based Powders and Their Gas-Sensing Properties as Thick Film Sensors

**DOI:** 10.3390/s110201261

**Published:** 2011-01-25

**Authors:** Luyang Yuan, Takeo Hyodo, Yasuhiro Shimizu, Makoto Egashira

**Affiliations:** 1 Graduate School of Science and Technology, Nagasaki University, 1-14 Bunkyo-machi, Nagasaki 852-8521, Japan; E-Mail: d708057c@cc.nagasaki-u.ac.jp (L.Y.); 2 Faculty of Engineering, Nagasaki University, 1-14 Bunkyo-machi, Nagasaki 852-8521, Japan; E-Mails: hyodo@nagasaki-u.ac.jp (T.H.); egashira@nagasaki-u.ac.jp (M.E.)

**Keywords:** mesopore, macropore, meso-macropore, SnO_2_ gas sensors, SiO_2_, Sb_2_O_5_

## Abstract

Mesoporous and/or macroporous SnO_2_-based powders have been prepared and their gas-sensing properties as thick film sensors towards H_2_ and NO_2_ have been investigated. The mesopores and macropores of various SnO_2_-based powders were controlled by self-assembly of sodium bis(2-ethylhexyl)sulfosuccinate and polymethyl-methacrylate (PMMA) microspheres (*ca*. 800 nm in diameter), respectively. The introduction of mesopores and macropores into SnO_2_-based sensors increased their sensor resistance in air significantly. The additions of SiO_2_ and Sb_2_O_5_ into mesoporous and/or macroporous SnO_2_ were found to improve the sensing properties of the sensors. The addition of SiO_2_ into mesoporous and/or macroporous SnO_2_ was found to increase the sensor resistance in air, whereas doping of Sb_2_O_5_ into mesoporous and/or macroporous SnO_2_ was found to markedly reduce the sensor resistance in air, and to increase the response to 1,000 ppm H_2_ as well as 1 ppm NO_2_ in air. Among all the sensors tested, meso-macroporous SnO_2_ added with 1 wt% SiO_2_ and 5 wt% Sb_2_O_5_, which were prepared with the above two templates simultaneously, exhibited the largest H_2_ and NO_2_ responses.

## 1. Introduction

In recent years, the development for porous materials is been an essential objective of materials science research. This interest is the result of the progress in all fields of industry and technology [1–5]. According to the IUPAC definition, microporous materials are those with pore diameters less than 2 nm, mesoporous materials are those that have pore diameters between 2 and 50 nm, and macroporous materials are those with pores bigger than 50 nm [6]. Among them, macroporous and mesoporous silica with sufficient thermal stability has been applied to catalysts [7,8] membranes [9], adsorbents [10], chemical sensors [11] and templates for nanowires [12]. On the contrary, the poor thermal stability of non-silica mesoporous materials limits their applications. Over the past 50 years, semiconductor metal oxides such as SnO_2_, ZnO and In_2_O_3_ have been extensively studied as gas sensing materials due to their various advantages such as the facile fabrication process of thin and thick films, low cost and high thermal stability [13,14]. Among the various metal oxides, SnO_2_ is one of the most attractive materials for semiconductor gas sensors [13–23] operated at elevated temperatures (200–600 °C). The gas sensing property of semiconductor gas sensors is largely dependent on various factors such as shape and size of the oxide particles [24–30]. In addition, strict control of nanostructure of the oxide powders is also quite effective in improving the gas sensing properties [31–34]. Thus, our group’s efforts have so far been directed to preparing thermally stable mesoporous (m-) [15–17] and macroporous (mp-) [18,21] oxide films. However, the H_2_ sensing properties of the m-SnO_2_ sensors were relatively lower than expected from their large specific surface area and mp-SnO_2_ showed rather excellent sensing properties to H_2_. Moreover, our recent studies have demonstrated the successful preparation of thermally stable meso-macroporous (m·mp-) SnO_2_ and the improvement of gas sensing properties by employing pellet-type sensor structures [19]. However, the mechanical strength of the m·mp-SnO_2_ pellets was not enough for long-term operation and this then became a subject for further investigation.

The present study is thus directed to developing m-, mp- and m·mp-SnO_2_ thick film sensors. The sensors were fabricated by screen-printing of their as-prepared powders, which were produced by employing sodium bis(2-ethylhexyl)sulfosuccinate (aerosol-OT, AOT) as a mesopore template and PMMA microspheres with an average diameter of 800 nm as a macropore template, and then subsequent calcination at 600 °C for 5 h. The effects of the addition of SiO_2_ and Sb_2_O_5_ to m-, mp- and m·mp-SnO_2_ powders on their H_2_ and NO_2_ sensing properties were also examined.

## 2. Experimental Section

### 2.1. Preparation of Mesoporous and/or Macroporous SnO_2_-Based Powders

Various SnO_2_-based powders with well-developed mesopores and/or macropores were prepared by a sol-gel method using SnCl_4_·5H_2_O (Kishida Chem. Co., Ltd.) as a Sn source, AOT (Kishida Chem. Co., Ltd.) as a mesopore template and PMMA microspheres with an average diameter of 800 nm (MP-1600, Soken Chem. & Eng. Co., Ltd.) as a macropore template. A given amount of SnCl_4_·5H_2_O (1.75 g) was mixed in 400 mL of ultra pure water together with an appropriate amount of AOT and/or PMMA microspheres. In some cases, appropriate amounts of tetraethoxysilane (TEOS, Kishida Chem. Co., Ltd.) and/or SbCl_3_ (Kishida Chem. Co., Ltd.) were also added to the solution, in order to prepare SnO_2_ powders added with the given amounts of SiO_2_ and/or Sb_2_O_5_. Then the pH value of the resulting mixture was adjusted to 8.5 by adding an aqueous solution of NH_3_. The solid product obtained was aged in the solution at 20 °C for 3 days, then the resulting product was separated from the solution by centrifugation. After drying the product in an oven at 80 °C overnight, the resulting powder product was treated with a 0.1 mol L^−1^ phosphoric acid solution for about 2 h, and the resulting product was dried in an oven at 80 °C overnight. The powder product resulting after pulverization is referred to as-prepared powder. The as-prepared powders were used for fabricating thick film sensors, but for the characterization tests, as-prepared powders were subjected to calcination at 600 °C for 5 h in air, which are the same conditions adopted for the thick film sensors after the screen-printing of a paste of as-prepared powders. The preparation conditions and compositions of all SnO_2_-based powders obtained in this study and their abbreviations are summarized in Table 1.

In our study, the SnO_2_-based powders prepared using AOT or PMMA microspheres as a template are identified by using abbreviations such as m-T*x*S*y* or mp-T*x*S*y*, respectively, and the SnO_2_-based powder prepared using both AOT and PMMA microspheres as templates is indicated as m·mp-T*x*S*y*, as shown in Table 1. Here, T and S mean the addition of TEOS and SbCl_3_ in the precursor solution, respectively, and *x* (*x* = 0, 1, 5) and *y* (*y* = 0, 1, 5) represent the added amounts of SiO_2_ and Sb_2_O_5_ (wt%) with respect to the weight of SnO_2_, respectively, on the basis of the expected weight of constituent oxides after calcination.

Crystal phase and crystallite size of SnO_2_-based powders were characterized with X-ray diffraction (XRD, CuKα, Shimadzu Corp., RINT-2200). The crystallite size was calculated by using Scherrer’s formula:

(1) CS = 0.89λ/βcosθ

where λ is the wavelength of CuKα, *β* is the full-width at the half-maximum of the (110) line and *θ* is the diffraction angle of the (110) peak. The specific surface area, pore volume and pore size distribution of SnO_2_-based powders were measured by the BET method using a N_2_ sorption isotherm (Micromeritics Instrument Corp., TriStar3000). Morphology of SnO_2_-based thick films was observed by a scanning electron microscope (SEM, JEOL Ltd., JCM-5700).

### 2.2. Fabrication of Mesoporous and/or Macroporous SnO_2_-Based Thick Film Sensors

The as-prepared SnO_2_-based powder was mixed with a printing oil which is composed of an alkyl ester of methacrylic acid as a binder, a toluene-based solvent, and an ammonium salt of polyacrylic acid as a plasticizer, and the resulting paste was screen printed on an alumina substrate, on which a pair of interdigitated Pt electrodes (gap between electrodes: 130 μm) had been printed (the thickness of the film was controlled to be about 20 μm after calcination for all the sensors fabricated). Then the printed film was subjected to heat treatment at 600 °C for 5 h in air prior to response measurements. The gas-sensing properties of the thick film sensors to 1,000 ppm H_2_ and 1 ppm NO_2_ balanced with air were measured in the temperature range of 350–500 °C. The magnitude of the gas response was defined as the ratio (R_a_/R_g_) of the sensor resistance in air (R_a_) to that in a target gas (R_g_) for H_2_, but the reverse ratio (R_g_/R_a_) was used for NO_2_.

## 3. Results and Discussion

### 3.1. Characterization of Mesoporous and/or Macroporous SnO_2_-Based Powders

**Figure 1.**
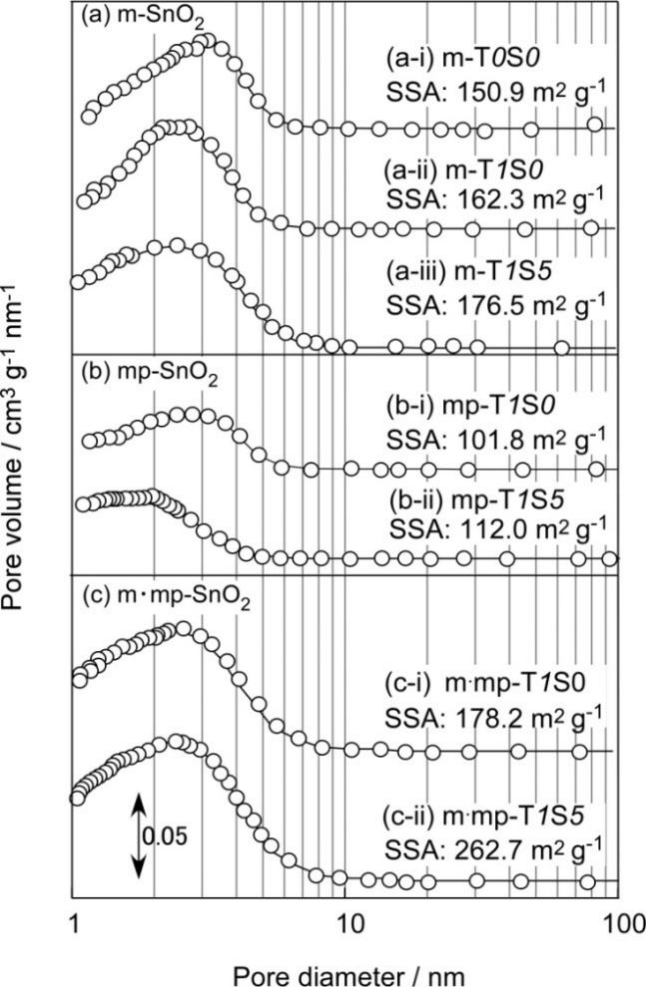
Pore size distributions and specific surface area of representative **(a)** m-SnO_2_, **(b)** mp-SnO_2_ and **(c)** m·mp-SnO_2_ powders.

Pore size distribution and specific surface area (SSA) of representative m-SnO_2_, mp-SnO_2_ and m·mp-SnO_2_ powders after calcination are shown in Figure 1. As shown in Figures 1(a-i), m-T*0*S*0* powder, which was prepared only with the addition of AOT, showed a SSA of 150.9 m^2^ g^−1^ and a larger pore volume of 0.153 cm^3^ g^−1^ with a pore diameter of ca. 3.1 nm at the maximum pore volume (hereafter, it will be referred to as the maximum pore diameter). The characterization data of representative SnO_2_-based powders is summarized in Table 2. The addition of 1 wt% SiO_2_ to m-T*0*S*0* induced a slight increase in SSA (162.3 m^2^ g^−1^) and reduced the maximum pore diameter to ca. 2.7 nm (see m-T*1*S*0*). This result implies the repression of growth of SnO_2_ crystallites and/or grains by the added SiO_2_, as was reported by Fukuoka *et al*. [12]. Simultaneous addition of 1 wt% SiO_2_ and 5 wt% Sb_2_O_5_ to m-T*0*S*0* resulted in further increase in SSA slightly to a value of 176.5 m^2^ g^−1^ (see m-T*1*S*5*). Thus, the addition of Sb_2_O_5_ was suggested to be also effective in controlling grain growth, which will be confirmed by the change in SnO_2_ crystallite size discussed later.

**Figure 2.**
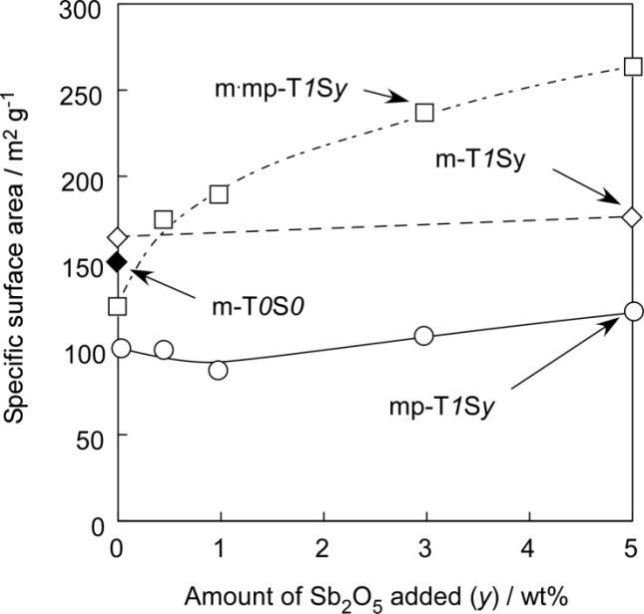
Variations in specific surface area of representative SnO_2_-based powders with amounts of Sb_2_O_5_ added.

It was revealed that the introduction of macropores into m-T*1*S*5* was very effective for increasing SSA to a value of 262.7 m^2^ g^−1^ (see m·mp-T*1*S*5*, Figures 1(c-ii)). This arises undoubtedly from the decrease in the maximum pore diameter and the increase in pore volume, as summarized in Table 2. On the other hand, the introduction of macropores only (mp-T*1*S*5*), instead of mesopores (m-T*1*S*5*), into SnO_2_-based powder reduced SSA to a value of 112.0 m^2^ g^−1^ (compare Figure 2(b-ii) with Figure 2(a-iii)) and then decreased pore volume (see Table 2). From these results, it is confirmed that the introduction of mesopores is essential for obtaining both large specific surface and large pore volume of SnO_2_-based powders.

Figure 2 shows variations in SSA of representative SnO_2_-based powders with amount of Sb_2_O_5_ added. The effect of the Sb_2_O_5_ on SSA can be seen more clearly from this figure. As for the cases of m-T*1*S*y* and mp-T*1*S*y* series, SSA values increased slightly with increasing amounts of Sb_2_O_5_ added, but only for the m·mp-T*1*S*y* series, it is obvious that SSA increased markedly with an increase in the additive amount of Sb_2_O_5_ reaching the largest value of 262.7 m^2^ g^−1^ obtained in the present study. The reason for this preferable effect for sensor application observed only the m·mp-T*1*S*y* series is not yet clarified and is a subject for future work.

**Figure 3.**
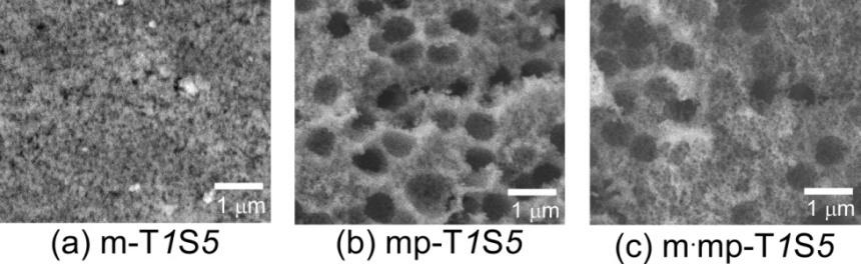
SEM images of fracture surface of **(a)** m-T*1*S*5*, **(b)** mp-T*1*S*5* and **(c)** m·mp-T*1*S*5* thick film sensors.

Figure 3 shows the SEM images of the fracture surface of m-T*1*S*5*, mp-T*1*S*5* and m·mp-T*1*S*5* thick film sensors. No formation of macropores in m-T*1*S*5* is reasonable, since no PMMA microspheres were added, as shown in Figure 3(a). But, Figure 3(b,c) confirm the formation of many spherical macropores originating from the morphology of PMMA microspheres as a template in the mp-T*1*S*5* and m·mp-T*1*S*5* thick film sensors. However, the diameter of macropores observed was in the 400–750 nm range, which was smaller than that of the diameter of raw PMMA microspheres, due to shrinkage of resulting voids during the growth of SnO_2_ crystallites.

**Figure 4.**
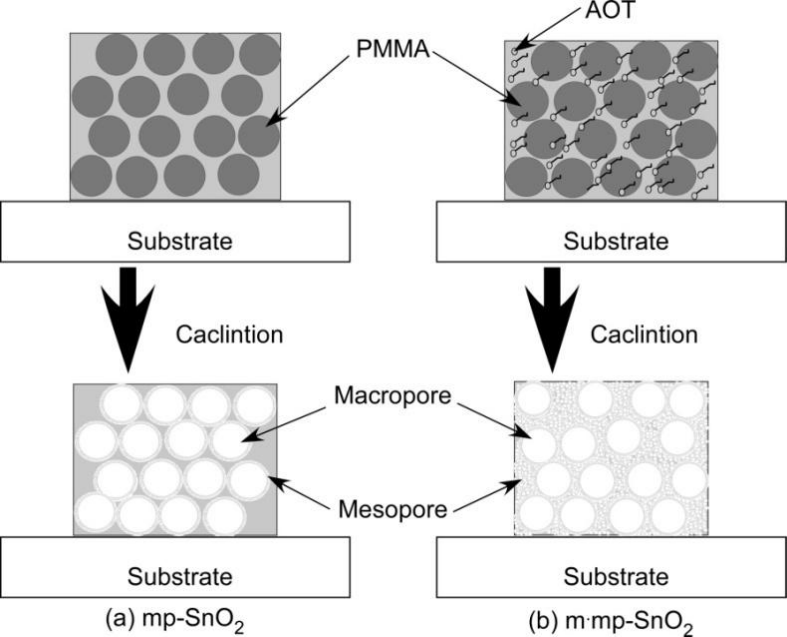
Schematic drawing of formation mechanism of mesopores and macropores in **(a)** mp-SnO_2_ and **(b)** m·mp-SnO_2_ thick film sensors.

As shown in Figure 1 and Table 1, mp-SnO_2_ powder prepared in the present study showed relatively larger SSA than the conventional SnO_2_ powder (8.4 m^2^ g^−1^, [35]), indicating the formation of a certain amount of mesopores, irrespective of the addition or not of AOT as a mesoporous structure template. This result implies penetration or diffusion of PMMA fragments into the dried SnO_2_ precursor material during the calcination and such fragments may act as a mesoporous template at the interface between the PMMA microsphere and surrounding dried SnO_2_ precursor. Thus, after the calcination at 600 °C for 5 h of the mp-SnO_2_ thick film, a thin mesoporous layer may be formed at the interface between the pores and SnO_2_ particles, as shown schematically in Figure 4(a) [36]. As for m·mp-SnO_2_, it is considered that mesopores are formed uniformly inside all the SnO_2_ particles and the whole thick film structure, as shown in Figure 4(b).

Another notable finding in Figure 3 is a relatively longer distance of the macropores in m·mp-T*1*S*5* than that in mp-T*1*S*5*. Since AOT was used as a mesoporous template in fabricating m·mp-SnO_2_ powder, thermal decomposition and subsequent firing along with generation of combustion gases may induce sponge and/or bulky structure with mesopores, leading to a longer distance of the macropores, as also shown schematically in Figure 4(b).

**Figure 5.**
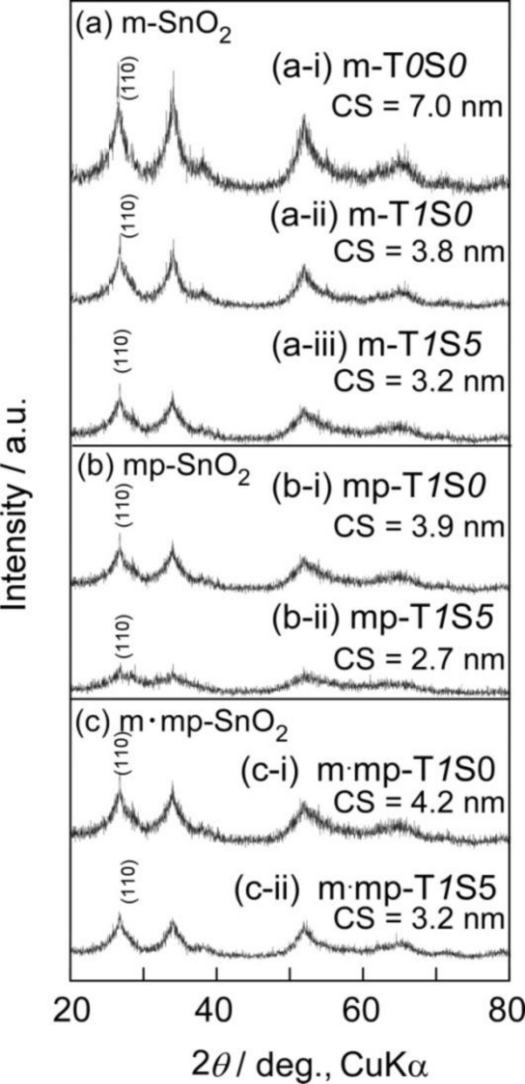
XRD patterns of **(a)** m-SnO_2_, **(b)** mp-SnO_2_ and **(c)** m·mp-SnO_2_ powders.

**Figure 6.**
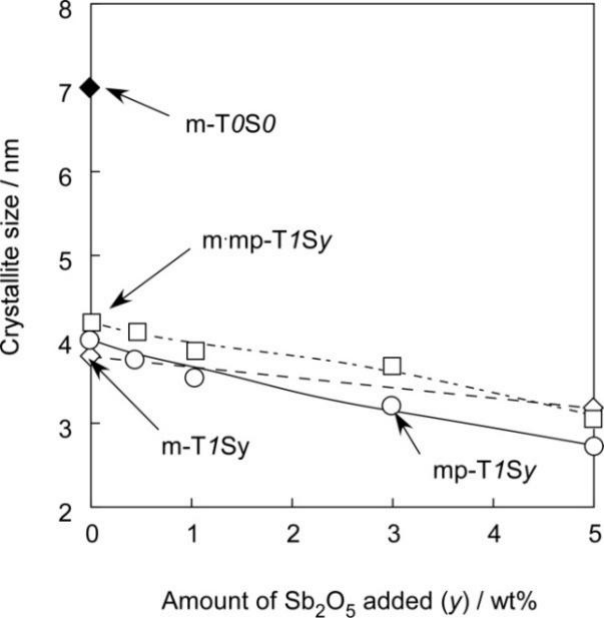
Variations in crystallite size of representative SnO_2_-based powders with amounts of Sb_2_O_5_ added.

Figure 5 shows XRD patterns of representative m-SnO_2_, mp-SnO_2_ and m·mp-SnO_2_ powders. Diffraction peaks of all powders were rather broad, indicating low crystallinity, but all peaks could be ascribed to those of tetragonal SnO_2_. The CS value which was calculated for each powder using Scherrer’s formula is summarized in Table 2. Variations in CS of representative SnO_2_-based powders are shown Figure 6. On the whole, the CS values were small and were in a range of 2.7–7 nm in diameter, due to the limitation of crystallite growth induced by the phosphoric acid treatment before the calcination [15,16]. Exceptionally, m-T*0*S*0* showed the largest CS value of 7 nm. The CS value was decreased drastically to 3.8 nm by the addition of 1 wt% SiO_2_ to m-T*0*S*0* (see m-T*1*S*0* in Table 2 and Figure 6). Thus, the repression of the growth of SnO_2_ crystallites by the added SiO_2_ could be confirmed from these results [37]. As for the powders containing 1 wt% SiO_2_, CS values were almost comparable, whereas they tended to decrease slightly with increasing amounts of the Sb_2_O_5_ additive in each series. In addition, the kind of porous structure, *i.e.*, mesopore, macropore, and mesopore plus macropore, was found to have only a little effect on controlling the CS values. Thus, we can confirm again that the addition of 1 wt% SiO_2_ was the most powerful method in reducing the CS value among several factors. The CS values decreased slightly by the Sb_2_O_5_ addition in each series, but no diffraction peaks other than SnO_2_ were observed in XRD patterns even for the cases of 5 wt% Sb_2_O_5_ addition (Figure 5). This implies that Sb ions added were sufficiently incorporated into the SnO_2_ crystal lattice and this solid-solution is also effective for the repression of the crystal growth among SnO_2_-based crystallites [23,38,39]. These results demonstrate that the pore size distribution, SSA and CS values of SnO_2_-based powders can be controlled by selecting the kinds of templates, the kind of additives and their additive amounts.

### 3.2. H_2_ and NO_2_ Sensing Properties of Mesoporous and/or Macroporous SnO_2_-Based Sensors

**Figure 7.**
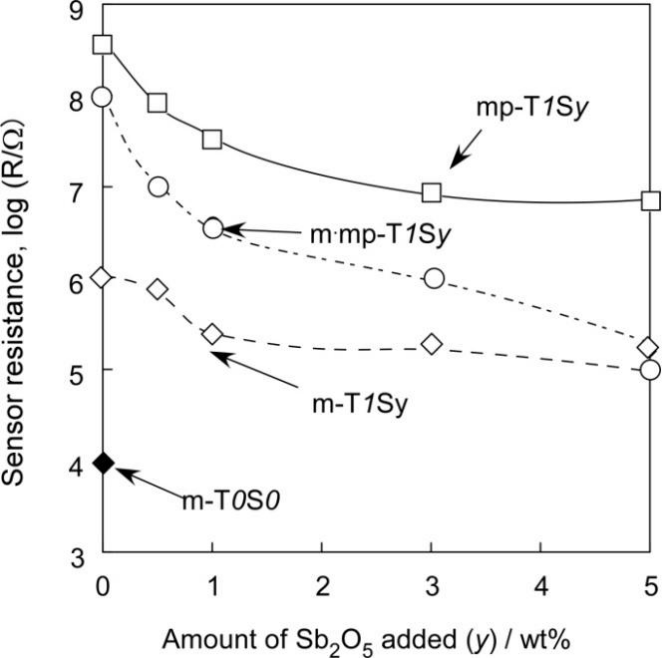
Variations in sensor resistance of SnO_2_-based thick film sensors in air at 450 °C with amounts of Sb_2_O_5_ added.

Variations in sensor resistance of SnO_2_-based thick film sensors in air at 450 °C with amounts of Sb_2_O_5_ added are shown in Figure 7. The m-T*0*S*0* sensor showed the lowest resistance in air, but the addition of 1 wt% SiO_2_ to m-T*0*S*0* increased the sensor resistance in air (see m-T*1*S*0*). The sensor resistance of other two series sensors, *i.e.*, mp-T*1*S*0* and m·mp-T*1*S*0*, in air was also very high. Even if Si^4+^ ions would be substituted for Sn^4+^ ion sites, no valency control effect could be expected. Therefore, SiO_2_ added was anticipated to be segregated among SnO_2_ crystallites and/or grains and then to reduce electronic conduction of SnO_2_-based thick film sensors, although the segregation of SiO_2_ was not confirmed by the XRD measurements due to its small amount added.

Introduction of macropores into SnO_2_ by using PMMA microspheres (see mp-T*1*S*y* series), instead of the introduction of mesopores, and/or the simultaneous introduction of macropores (see m·mp-T*1*S*y* series) also resulted in an increase in sensor resistance. This phenomenon can be considered to arise mainly from the introduction of air voids, which are electrical insulators, via various pores in the thick film sensors, but the mp-T*1*S*y* sensor with macropores showed the largest resistance in air, irrespective of the smallest pore volume, among three series of sensors. This fact implies the existence of another factor, besides the pore volume, in determining the sensor resistance in air, such as the manner of distribution of pores in the thick film and so on, though the details are not clear at present.

In each sensor series, the sensor resistance in air decreased with increasing amounts of Sb_2_O_5_ additive. This behavior can be explained by the valency control, *i.e.*, partial substitution of Sn^4+^ sites with Sb^5+^ ions, producing free electrons, as described in Equation (2) [38–40]:

(2) $${\text{Sb}}_{2}O_{5}\to 2\;{\text{Sb}}_{\text{Sn}}+4\;{O_{O}}^{x}+1/2\;O_{2}\;(g)+2\;e'$$

These results also confirm the existence of substituted Sb^5+^ ions, *i.e.*, the solid-solution of between Sb_2_O_5_ and SnO_2_ and then little amount of segregated Sb_2_O_5_ among SnO_2_-based particles.

**Figure 8.**
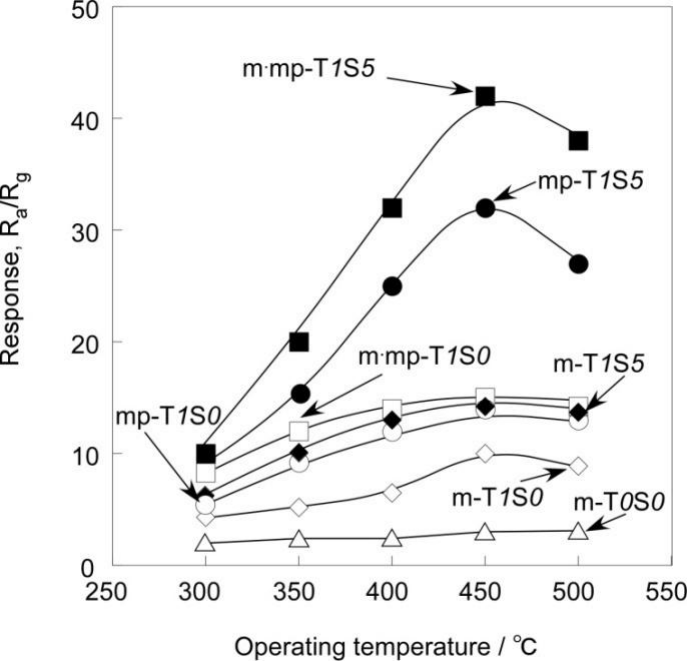
Temperature dependence of response SnO_2_-based thick film sensors to 1,000 ppm H_2_.

**Figure 9.**
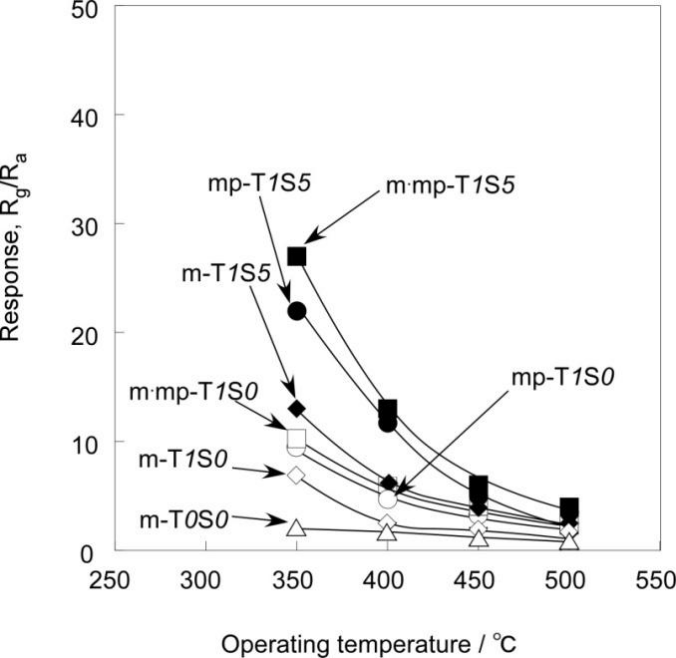
Temperature dependence of response SnO_2_-based thick film sensors to 1 ppm NO_2_.

Figures 8 and 9 show temperature dependence of response of SnO_2_-based thick film sensors to 1,000 ppm H_2_ balanced with air and 1 ppm NO_2_ balanced with air. Almost all sensors showed the maximum response to 1,000 ppm H_2_ at a temperature of 450 °C. In contrast, the response to 1 ppm NO_2_ of all sensors tended to increase as the operating temperature decreased, and showed the largest response in the temperature range studied at 350 °C.

**Figure 10.**
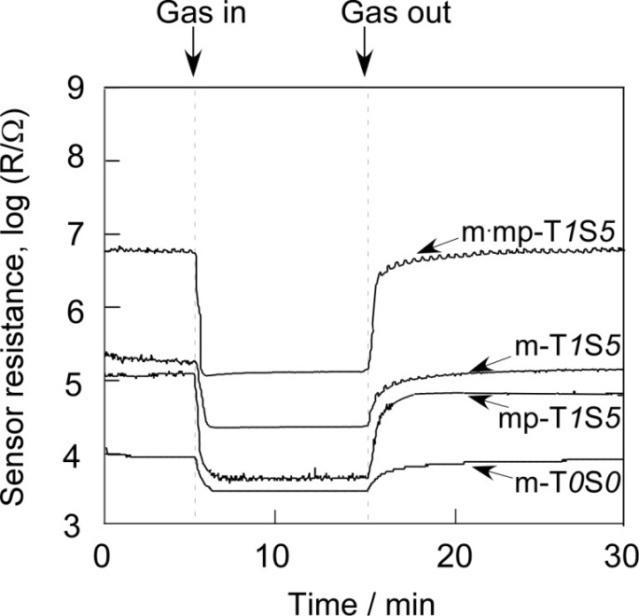
Response transients of SnO_2_-based thick film sensors to 1,000 ppm H_2_ in air at 450 °C.

**Figure 11.**
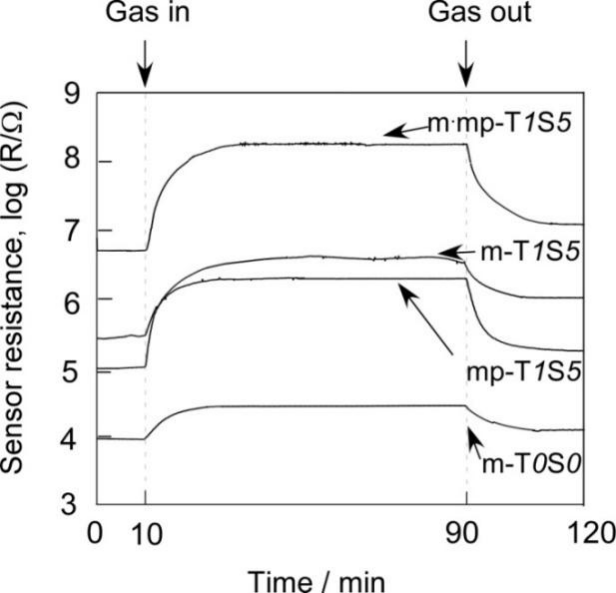
Response transients of SnO_2_-based thick film sensors to 1 ppm NO_2_ in air at 350 °C.

Response transients of SnO_2_-based thick film sensors to 1,000 ppm H_2_ at 450 °C and 1 ppm NO_2_ at 350 °C balanced with air are shown in Figures 10 and 11, respectively. In this study, 50% response time is defined as a period necessary to achieve 50% of resistance value of R_a_ − R_g_, while 50% recovery time is defined as that necessary to achieve 50% of resistance value of R_g_ − R_a_ for H_2_. The 50% response and recovery times to NO_2_ are also defined in the similar manner, but by using R_g_ − R_a_ for response time and R_a_ − R_g_ for recovery time. Hereafter, they are simply expressed as response time and recovery time, respectively. Response and recovery times of SnO_2_-based thick film sensors to 1,000 ppm H_2_ at 450 °C and 1 ppm NO_2_ at 350 °C were summarized in Table 3. The m-T*0*S*0* sensor showed the longest response and recovery times to H_2_ among the sensors listed in Table 3. The simultaneous addition of 1 wt% SiO_2_ and 5 wt% Sb_2_O_5_ to m-T*0*S*0* was found to shorten slightly response and recovery times to H_2_ (see m-T*1*S*5*).

However, the m-T*1*S*5* sensor showed longer response and recovery times to 1 ppm NO_2_ than m-T*0*S*0*. Thus, the effect of additive on the response and recovery times varied with the kind of target gas. The introduction of macropores into m-T*1*S*5* shortens the response and recovery times to H_2_. More remarkable shortening of the recovery time to H_2_ as well as response and recovery times to NO_2_ were observed with m·mp-T*1*S*5*. It is reasonable to consider that the response time to H_2_ is closely related to the diffusivity of H_2_, while the recovery time is controlled by the diffusivity of O_2_ which has a larger molecular size than H_2_. As for NO_2_, on the other hand, both the response and recovery times are considered to affected by the diffusivity of NO_2_ itself, which has a larger molecular size than H_2_, from its gas sensing mechanism. Such considerations predict a shorter recovery time to H_2_ as well as shorter response and recovery times to NO_2_ by the introduction of macropores into the sensor materials. The results obtained with m·mp-T*1*S*5* were in good agreement with this prediction. Thus, the mp-T*1*S*5* sensor, which was fabricated only by the introduction of macropores, showed the fastest response to H_2_ as well as the fastest response and recovery times to NO_2_ among the sensors tested. But, the reason for the longer recovery time to H_2_ of mp-T*1*S*5* than m·mp-T*1*S*5* is not clear at present. Anyway, such behavior undoubtedly arises from more easy diffusion of a target gas as well as oxygen through mesopores rather than macropores. On the other hand, all of the response and recovery times to NO_2_ are much longer than those to H_2_. This may arise not only from slow diffusivity of NO_2_ in comparison to H_2_, but also from slow adsorption rate and strong interaction of NO_2_^−^ species on the oxide surface.

**Figure 12.**
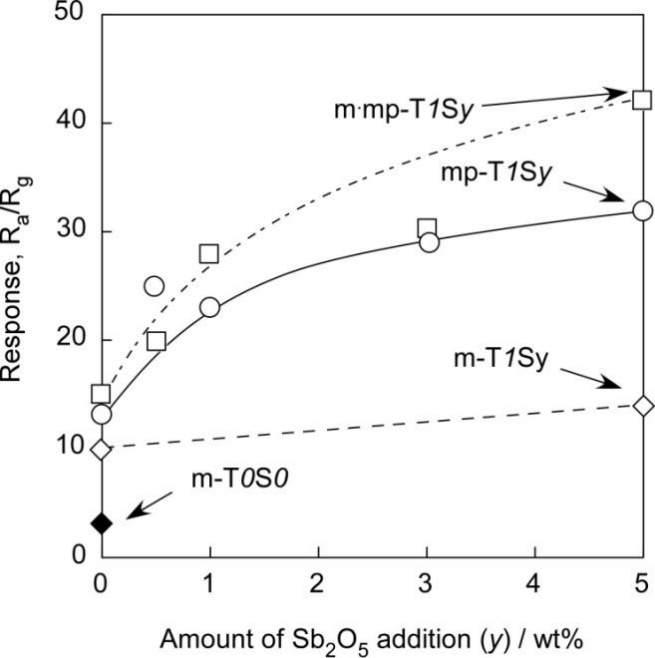
Variations in response of SnO_2_-based sensors to 1,000 ppm H_2_ in air at 450 °C with amounts of Sb_2_O_5_ added.

**Figure 13.**
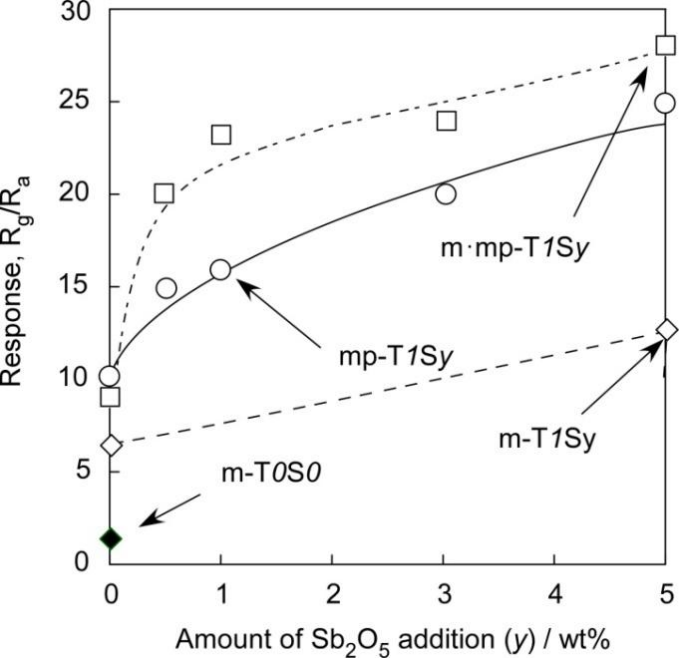
Variations in response of SnO_2_-based sensors to 1 ppm NO_2_ in air at 350 °C with amounts of Sb_2_O_5_ added.

Figures 12 and 13 show variations in responses of SnO_2_-based sensors to 1,000 ppm H_2_ at 450 °C and to 1 ppm NO_2_ at 350 °C in air with amounts of Sb_2_O_5_ added, respectively. From these figures, it is also apparent that the m-T*0*S*0* sensor showed the smallest responses to both H_2_ and NO_2_ among the sensors studied. The addition of 1 wt% SiO_2_ to m-T*0*S*0* enhanced responses to both H_2_ and NO_2_ to a certain level for every series of sensors. In addition, H_2_ and NO_2_ responses increased with increasing amounts of Sb_2_O_5_ additive in each series of sensors. On the whole, the magnitude of the response was in the order of m·mp-T*1*S*y* > mp-T*1*S*y* > m-T*1*S*y*, when the comparison was made at the same additive amount of Sb_2_O_5_, with only one exception observed for the NO_2_ response of the m·mp-T*1*S*0* sensor. It is worth noting that the mp-T*1*S*y* series sensors showed higher H_2_ and NO_2_ responses than those of m-T*1*S*y* series sensors, irrespective of their smaller surface area. This implies that all the surface of sensor materials including the inner surface of mesopores is not utilized effectively for gas detection, and that easy diffusion of a target gas as well as oxygen to the active surface, *i.e.*, the existence of certain amounts of macropores inside the thick film sensors, is more important for improving gas response. The highest H_2_ and NO_2_ responses observed with the m·mp-T*1*S*y* sensors may be a result of good combination of mesopore and macropores in the thick film sensors. From these results, it was revealed that the strict control of microstructure having well-developed mesoporous and macroporous is indispensable to enhancing gas reactivity and diffusivity and thus to improving responses to H_2_ and NO_2_ in air.

## 4. Conclusions

Mesoporous and/or macroporous SnO_2_-based powders have been prepared by a sol-gel method by employing SnCl_4_·5H_2_O, ATO as a mesopore template, PMMA microspheres as a macropore template, and their gas-sensing properties as thick film sensors towards 1 ppm NO_2_ as well as 1,000 ppm H_2_ in air have been investigated. The addition of SiO_2_ into mesoporous and/or macroporous SnO_2_ was found to increase SSA of mesoporous SnO_2_. However, the SSA of all samples increased and their CS tended to decrease slightly with the addition of the Sb_2_O_5_. The additions of SiO_2_ and Sb_2_O_5_ into mesoporous and/or macroporous SnO_2_ were found to improve the sensing properties of the resulting sensors. The addition of SiO_2_ into mesoporous and/or macroporous SnO_2_ was found to increase the sensor resistance in air. However, the doping of Sb_2_O_5_ into mesoporous and/or macroporous SnO_2_ was found to markedly reduce the sensor resistance in air, and to increase the response to 1,000 ppm H_2_ as well as 1 ppm NO_2_ in air. Among all the sensors tested, meso-macroporous SnO_2_ mixed with 1 wt% SiO_2_ and 5 wt% Sb_2_O_5_, which were prepared with above two templates simultaneously, exhibited the largest H_2_ and NO_2_ responses.

## Figures and Tables

**Table 1.** Preparation conditions of SnO_2_-based powders.

**Sensors**		Mesopore template (AOT) /g*	Macropore template (PMMA) /g*	**Amount of MO added to SnO_2_ (*x* or *y*)/wt%**	
**Kind of powder**	**Abbreviation**			**MO: Sb_2_O_5_ (using NbCl_5_)**	**MO: SiO_2_ (using TEOS)**
**Mesoporous (m-) SnO_2_**	m-T*0*S*0*	1.75	none	none	none
	m-T*1*S*0*				1.0
	m-T*1*S*5*			5.0	

**Meso-macroporous (m·mp-) SnO_2_**	m·mp-T*1*S*0*	1.75	0.35	none	1.0
	m·mp-T*1*S*0.5*			0.5	
	m·mp-T*1*S*1*			1.0	
	m·mp-T*1*S*3*			3.0	
	m·mp-T*1*S*5*			5.0	

**Macroporous (mp-) SnO_2_**	mp-T*1*S*0*	none	0.35	none	1.0
	mp-T*1*S*0.5*			0.5	
	mp-T*1*S*1*			1.0	
	mp-T*1*S*3*			3.0	
	mp-T*1*S*5*			5.0	

* In 400 mL aqueous solution.

**Table 2.** Characterization data of representative m-SnO_2_, mp-SnO_2_ and m·mp-SnO_2_ powders.

**Sensors**		**Specific surface area (SSA) /m^2^ g^−1^**	**Pore volume/cm^3^ g^−1^**	Maximum pore diameter */nm	**Crystallite size (CS)/nm**
**Kind of powder**	**Abbreviation**				
**m-SnO_2_**	m-T*0*S*0*	150.9	0.153	3.1	7.0
	m-T*1*S*0*	162.3	0.160	2.7	3.8
	m-T*1*S*5*	176.5	0.155	2.5	3.2

**m·mp-SnO_2_**	m·mp-T*1*S*0*	178.2	0.184	2.5	4.2
	m·mp-T*1*S*5*	262.7	0.191	2.3	3.2

**mp-SnO_2_**	mp-T*1*S*0*	101.8	0.090	2.9	3.9
	mp-T*1*S*5*	112.0	0.079	2.0	2.7

* Pore diameter at the maximum pore volume in the pore size distribution curve.

**Table 3.** 50% response time and 50% response time of SnO_2_-based thick film sensors to 1,000 ppm H_2_ at 450 °C and 1 ppm NO_2_ at 350 °C balanced with air.

**Sensors**		**1,000 ppm H_2_ (450 °C)**		**1 ppm NO_2_ (350 °C)**	
**Kind of powder**	**Abbreviation**	**50% response time/s**	**50% recovery time/s**	**50% response time/s**	**50% recovery time/s**
**m-SnO_2_**	m-T*0*S*0* m-T*1*S*5*	25 22	35 29	182 195	330 600
**m·mp-SnO_2_**	m·mp-T*1*S*5*	20	17	154	325
**mp-SnO_2_**	mp-T*1*S*5*	16	22	110	220
